# De Novo Sporophyte Transcriptome Assembly and Functional Annotation in the Endangered Fern Species *Vandenboschia speciosa* (Willd.) G. Kunkel

**DOI:** 10.3390/genes12071017

**Published:** 2021-06-30

**Authors:** Mohammed Bakkali, Rubén Martín-Blázquez, Mercedes Ruiz-Estévez, Manuel A. Garrido-Ramos

**Affiliations:** 1 Departamento de Genética, Facultad de Ciencias, Universidad de Granada, 18071 Granada, Spain; mbakkali@ugr.es (M.B.); rumabl@illinois.edu (R.M.-B.); mercedesruizestevez@gmail.com (M.R.-E.); 2 Department of Entomology, University of Illinois Urbana-Champaign, Urbana, IL 61801, USA; 3 Recombinetics Incorporated, 3388 Mike Collins Drive, Eagan, MN 55121, USA

**Keywords:** transcriptome, functional annotation, ferns, *Vandenboschia speciosa*

## Abstract

We sequenced the sporophyte transcriptome of Killarney fern (*Vandenboschia speciosa* (Willd.) G. Kunkel). In addition to being a rare endangered Macaronesian-European endemism, this species has a huge genome (10.52 Gb) as well as particular biological features and extreme ecological requirements. These characteristics, together with the systematic position of ferns among vascular plants, make it of high interest for evolutionary, conservation and functional genomics studies. The transcriptome was constructed de novo and contained 36,430 transcripts, of which 17,706 had valid BLAST hits. A total of 19,539 transcripts showed at least one of the 7362 GO terms assigned to the transcriptome, whereas 6547 transcripts showed at least one of the 1359 KEGG assigned terms. A prospective analysis of functional annotation results provided relevant insights on genes involved in important functions such as growth and development as well as physiological adaptations. In this context, a catalogue of genes involved in the genetic control of plant development, during the vegetative to reproductive transition, in stress response as well as genes coding for transcription factors is given. Altogether, this study provides a first step towards understanding the gene expression of a significant fern species and the in silico functional and comparative analyses reported here provide important data and insights for further comparative evolutionary studies in ferns and land plants in general.

## 1. Introduction

The family Hymenophyllaceae has its origin in the Triassic and currently contains about 600 species, most of which emerged approximately 160 million years ago during the Jurassic period [1,2] and show a great diversity in morphology and habitat occupation [3,4]. While other species of this family have a current pantropical distribution, the species object of the current study, *Vandenboschia speciosa* (Willd.) G. Kunkel (=*Trichomanes speciosum* Willd.), is a tetraploid species (2*n* = 144 chromosomes; C-value = 10.52 Gb) [4,5,6,7,8] that constitutes a rare Macaronesian-European endemism. Additionally, it is the only representative in the Macaronesian-European area of a genus of mainly tropical distribution. The ecological requirements of this species explain its current distribution, restricted disjunctively to the European Atlantic stripe and the Macaronesian islands (Azores, Madeira, and the Canary Islands). Its populations are found in places considered refuges of tertiary flora, suggesting their relic nature after the glacial cycles that occurred during the Tertiary period. *V. speciosa* is considered one of the most vulnerable fern species in Europe since it is threatened by habitat destruction and excessive collection and is listed in Annex I of the Berne Convention and Annex II of the Habitats Directive. At the national level, it is considered vulnerable in the Red List of Spanish Vascular Flora 2000. The two phases of its life cycle are perennial and capable of reproducing by vegetative propagation [9]. The sporophyte is rhizomatous and capable of propagating by fragmentation of its rhizome. Characteristically, fronds are constituted by translucent leaves composed of a single layer of cells, thus having little control over water loss [9,10,11]. The species requires constantly humid and warm winter conditions, which restricts the species to extreme low-light environments [9,10,11]. The gametophyte, very different from the typical heart-shaped prothallus, it is epigeous and narrowly filamentous (to such an extent that it is often confused with the protonema of a bryophyte). While the sporophyte is adapted to grow in areas with a low incidence of light and constant humidity, the gametophyte can live in a wider range of habitats, including those that are darker and less humid. Such sites can provide a microclimate and a stable environment for long-term survival of independent gametophytes outside the sporophyte distribution range [9]. The adaptive responses allowing life in such conditions could be facilitated by morphological and physiological changes in the gametophyte [10,11]. A mechanism consisting in the production of asexual propagules, called gemmae [11], has evolved in this and in a few other species to perpetuate the gametophyte by vegetative propagation, without intervention of the sporophyte, in some populations [9,12,13,14,15]. Indeed, in several populations of species such as *Vittaria appalachiana*, *Hymenophyllum tayloriae* and *Vandenboschia intrincatum*, the sporophytic phase of the life cycle has been completely eliminated, with the gametophyte surviving for a great length of time [12,13,14,15]. 

A comprehensive transcriptome analysis in *V. speciosa* would represent an important scientific resource for gene discovery and functional genomics studies. These studies might shed light on the knowledge of the genetic regulation of the adaptive response of both the sporophyte and the gametophyte and could be of use on the conservation genetics of this and similar species. In this context, the identification of the genes that regulate the growth and development of both phases of this species is also of special importance as it entails learning the genetic control of the transition between the vegetative and reproductive phases and the alternation between sporophyte and gametophyte. Here, we provide the de novo assembly and characterization of the sporophyte transcriptome of this vulnerable species, which represents an important step towards understanding the gene expression associated with phenotypes in a species with singular sporophytes and gametophytes, extreme ecological requirements and populations composed of independent gametophytes. In addition, the sporophyte transcriptome data and the functional analyses reported here provide an important platform for comparative evolutionary studies in ferns and land plants in general.

## 2. Materials and Methods

### 2.1. Sample Collection

*V. speciosa* sporophytes were collected in April 2014 at one out of the seven populations located in the Alcornocales Natural Park (Cádiz, Spain): Valdeinfierno (VDI). We chose a population with a regular fern lifecycle of two free-living generations, gametophyte, and sporophyte. Sporophytes were frozen in liquid nitrogen in the field and stored at −80 °C. 

### 2.2. Next Generation Sequencing and Transcriptome Assembly

RNA was isolated from five sporophyte specimens using Spectrum™ Plant Total RNA Kit (Sigma, Madrid, Spain). RNAs were pooled and Next Generation Sequencing was carried out at Macrogen Inc. (Macrogen Inc., Seoul, Korea) based on the Illumina HiSeq 2000 Paired-end approach (Illumina Inc., Seoul, South Korea). Illumina sequencing data can be accessed at Short Read Archive (SRA) ENA database under the accession number ERX2079928.

Quality assessment of the resulting sequenced reads was performed using FastQC (https://www.bioinformatics.babraham.ac.uk/projects/fastqc/; accessed on 10 April 2019). De novo transcriptome assembly was performed using Trinity v2.8 [16] as follows: Trinity --trimmomatic--seqType fq--max_memory 50G--left filename_1.fastq--right filename_2.fastq--CPU 6. The option--trimmomatic enables quality trimming of reads using Trimmomatic [17] under default settings [18]. In silico normalization of the total reads was according to Trinity v2.8 defaults (defaults to max. read coverage of 200). Assembly statistics were computed using the script TrinityStats.pl contained in the Trinity package as well as with TransRate [19]. Furthermore, we applied the E90N50 statistic, which is an alternative to the Contig N50 statistic, and whose N50 statistic is limited to the top most highly expressed genes that represent 90% of the total normalized expression data. This calculation requires transcript abundance estimation (see Section 2.5) and is computed using the Trinity script contig_ExN50_statistic.pl. Further quality assessment of the assembled transcriptome was carried out following two additional procedures. One was based on examining the number of assembled transcripts that appear to be full-length or nearly full-length—Trinity carries out this analysis based on BLAST+ [20] searching on the SwissProt database [21] followed by examination of the percent of the target length that aligns to the best matching transcript, using the script analyze_blastPlus_topHit_coverage.pl. The second method relied on Bowtie2 [22] assessment of the proportion of reads that mapped to the assembled transcripts.

### 2.3. Assembly Filtering

The transcripts were filtered in order to reduce the probability of obtaining spurious transcripts and to attenuate transcript redundancy. Low expressed transcripts were removed based on their expression values as recommended by Trinity [16]. Briefly, transcripts per million (TPM) values, used as transcript abundance estimators, were obtained by the RSEM method [23] using the Trinity script aling_and_estimate_abundance.pl and the trimmed reads. Low expressed transcripts (TPM < 1) were then removed using the Trinity script filter_low_expr_transcripts.pl. A second filtering step was conducted to purge redundant transcripts and to generate non-redundant representative transcripts using CD-HIT-EST [24] with an identity threshold of 95%. The resulting FASTA file was used for subsequent analyses.

### 2.4. Assembly Completeness

We measured the completeness of the filtered transcriptome assembly using BUSCO v. 4.1.4 [25] through Galaxy Europe at https://usegalaxy.eu/ (accessed on 10 February 2021) [26]. BUSCO software employs sets of benchmarking universal single-copy orthologs from OrthoDB (www.orthodb.org; accessed on 10 February 2021) to provide quantitative measures of the completeness of transcriptome assemblies in terms of expected gene content. We analyzed the percentage of conserved single-copy orthologues represented in three datasets (eukaryotes, green plants, and land plants), using the OrthoDB sets: Eukaryota_odb10, Viridiplantae_odb10 and Embryophyta_odb10. Plotting of the BUSCO results was performed using the script generate_plot.py—provided as companion script to BUSCO (https://busco.ezlab.org/busco_userguide.html#companion-scripts; accessed on 17 February 2021) that uses ggplot2 [27] in R [28].

### 2.5. Transcript Abundance

We estimated transcript abundance in a genome-free manner (i.e., by aligning reads to the transcript assembly) with the “align_and_estimate_abundance” Perl script, using bowtie2 [22] for the alignment and RSEM as the abundance estimation method [23]. We thus obtained estimates of the count of reads that were derived from each transcript and then built a transcript expression matrix using the “abundance_estimates_to_matrix.pl” script to generate a normalized expression values matrix that was used to obtain the expression level of each transcript by ExN50 analysis. The normalized measure of each transcript expression was estimated as ‘fragments per kilobase transcript length per million fragments mapped’ (FPKM) and ‘transcripts per million transcripts’ (TPM) [23,29]. 

### 2.6. Coding Regions Identification

Candidate coding domain sequences (cds) within transcript sequences were identified with TransDecoder [16] using default parameters (minimum cut-off of 100 amino acids). To further maximize sensitivity for capturing ORFs that may have functional significance, regardless of the coding likelihood score criterion used by TransDecoder, we scanned all ORFs for homology to known proteins and retained all such ORFs for a BLAST search against SwissProt database and a PFAM search to identify common protein domains.

### 2.7. Functional Annotation

We used BLAST2GO software [30] for functional annotation. BLAST2GO uses the Basic Local Alignment Search Tool (BLAST) [31] to find sequences similar to the query set. The resulting FASTA file with sporophyte transcripts of *V. speciosa* generated after assembly and filtering was analyzed with BLASTx [32] against the nr database (NCBI non-redundant protein sequences, https://www.ncbi.nlm.nih.gov/; accessed on 27 July 2020). Following the recommended default parameters [30,33], BLASTx hits were considered positive at a 10^−3^ e-value threshold. BLAST annotation was exported as a FASTA file to generate an annotated transcript database. The resulting database was deposited in FigShare (https://figshare.com/; doi:10.6084/m9.figshare.14827956; accessed on 23 June 2021). Gene Ontology (GO) terms (http://www.geneontology.org; accessed on 8 October 2020) associated with the obtained BLAST hits were retrieved and GO annotation was carried out with BLAST2GO (*e*-value hit filter: 10^−6^). In addition, InterPro annotations in BLAST2GO allowed retrieval of domain/motif information in a sequence-wise manner. Corresponding GO terms associated to InterPro results were then transferred to the sequences and merged with already existing GO terms. Augmented annotation by Annex was carried out. BLAST2GO integrates the second layer concept developed by the Norwegian University of Science and Technology [34] for augmenting GO annotation. Enzyme code (EC) annotation was also carried out using BLAST2GO.

### 2.8. Gene Orthology Prediction

We used Orthofinder [35,36] to identify orthologous groups (orthogroups) of protein sequences among *V. speciosa* and a selected group of the following land plant species: the mosses *Ceratodon purpureus* (Hedw.) Brid. (https://www.ncbi.nlm.nih.gov/genome/12864; accessed on 17 March 2021) and *Physcomitrella patens* (Hedw.) Bruch. and Schimp. (http://www.plantgdb.org/XGDB/phplib/download.php?GDB=Pp; accessed on 12 February 2021), the ferns *Azolla filiculoides* Lam. and *Salvinia cucullata* Roxb. ex Bory (https://ftp.fernbase.org/; high-confidence genes; accessed on 12 February 2021) as well as *Ceratopteris richardii* Brongn. (https://www.datadepot.rcac.purdue.edu/jwisecav/genomes/Ceratopteris/Hn-n-denovo-transcriptome_v1.0/; accessed on 17 March 2021), the lycophyte *Selaginella moellendorffii* Hieron. (http://www.plantgdb.org/XGDB/phplib/download.php?GDB=Sm; accessed on 12 February 2021), and the seed plant *Arabidopsis thaliana* (L.) Heynh., (https://www.arabidopsis.org/download/index-auto.jsp?dir=%2Fdownload_files%2FSequences%2FAraport11_blastsets; accessed on 12 February 2021). Using reciprocal best-hits by the BLAST all-v-all algorithm, Orthofinder determined the number of shared putative orthologues between the eight species as well as species-specific transcripts. Orthofinder also infers gene trees for all orthogroups and a rooted species tree for the species being analyzed. Phylograms were visualized with Dendroscope [37].

## 3. Results and Discussion

### 3.1. Sequencing Outputs and De Novo Transcriptome Assembly

Sequencing statistics are displayed in Table 1. We obtained about 66.3 million paired-end sequencing reads of 101 bp of length (about 6700 million bases). After quality filtering with Trimmomatic, 98.35% of the paired-end reads were retained (65,249,598 reads and 6,590,209,398 bases). This high proportion of reads retained after quality trimming suggests that the library was of sufficient quality to obtain a high coverage of good-quality reads. 

De novo transcriptome assembly statistics are shown in Table 2. About 97 million bases were assembled into 84,759 transcripts, with median and average transcript lengths of 722 and 1,144.86 nucleotides, respectively. Fifty percent of the assembled bases were incorporated in transcripts of at least 1955 nucleotides in length. The Ex90N50 statistic was 2039 with 14,645 transcripts corresponding to the Ex 90 peak. The overall alignment rate of paired-end reads to reference was 95.35%. 8875 transcripts matched with a protein from the Swiss Prot database: 3301 of them (37.2% of transcripts) were covered by more than 90% of their target protein length and 6744 (76% of transcripts) were covered by more than 50% of their target protein lengths (Table 3). Taken together, all these results represent a first indication of a good quality assembly.

### 3.2. Transcripts Filtering and Assembly Completeness

To reduce the number of potential spurious transcripts, the assembly was filtered based on minimum transcript expression levels. Using a stringency of a minimum of 1 TPM, we kept 46,248 transcripts (54.56% of the initially assembled transcripts). Redundancy was further eliminated by clustering the filtered assembled transcripts using CD-HIT-EST at a nucleotide identity of 95%. Fewer than 22% of the transcripts were redundant and were therefore removed. The final filtered assembly contains 36,430 transcripts, with median and average transcript lengths of 1197 and 1437.37 nucleotides, respectively (Table 2). Fifty percent of the assembled bases were incorporated in transcripts of at least 2085 nucleotides in length (Table 2). These measures of transcripts contiguity were higher than those obtained in similar plant projects [38,39,40,41], including the fern species *Polypodium amorphum* Suksd. [42] and were comparable to those obtained for the transcriptome of *Prunus salicina* Lindl. [43], although lower than that of the fern species *Ceratopteris richardii* [44]. The N50 value limited to the top most highly expressed genes that represent 90% of the total normalized expression data (Ex90N50) was 2299, with 21,543 transcripts corresponding to the Ex 90 peak (Table 2). The final transcriptome was assembled based on 93.57% of the sequencing reads. *V. speciosa* transcripts gave identity to 7851 proteins of which 3196 (40.7% of transcripts) were covered in more than 90% of their lengths and 6444 (82% of transcripts) were covered in more than 50% of their lengths (Table 3).

**Figure 1:**
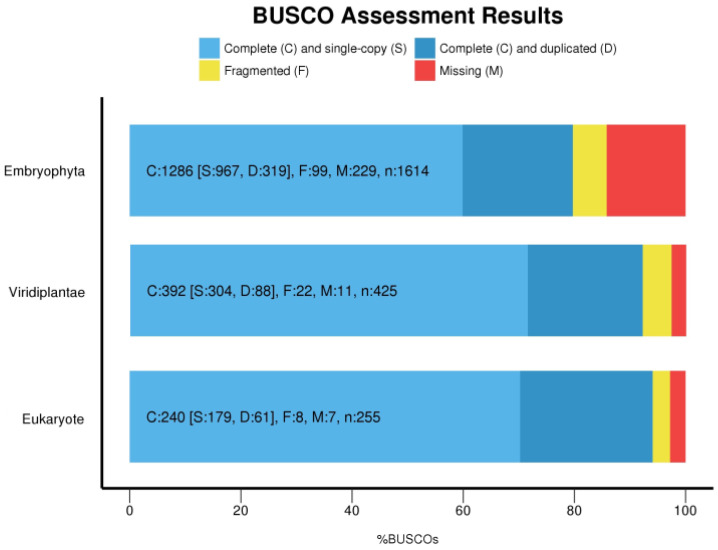
BUSCO completeness assessments of the *V. speciosa* filtered transcriptome with the Eukaryote (*n* = 255 conserved genes), Viridiplantae (*n* = 425 conserved genes) and Embryophyta (*n* = 1614 conserved genes) datasets. Blue, yellow, and red bars, respectively, represent the proportion of complete (C), fragmented (F), and missing (M) BUSCO genes. Light blue bars represent complete and single-copy BUSCO genes (S). Dark blue bars represent complete and duplicated BUSCO genes (D). Numbers within bars are absolute numbers of recovered (complete or fragmented) and missing genes. For example, for Embryophyta, the total dataset is composed of *n* = 1614 genes, of which 1286 (79.7%) were complete, 99 (6.1%) were fragmented, and 229 (14.2%) were not recovered. Among the complete genes, 319 represented duplicated genes and 967 represented single-copy genes.

Completeness of the filtered transcriptome assembly was measured using BUSCO [25]. A total of 94.1% of the 255 orthologues searched in the eukaryote ortholog sequence set were recovered completely (23.9% were duplicates) with another 3.1% represented as partial sequences (Figure 1). A total of 92.2% (20.7% duplicated) of the 425 orthologues searched in the green plant lineage were completely recovered with another 5.2% represented as partial sequences (Figure 1), and among the 1,604 orthologues searched in the land plant lineage, 79.7% were recovered completely (19.8% putative paralogues or duplicated) with another 6.1% represented as partial sequences (Figure 1). The percentage of duplicated sequences and paralogues was higher when the completeness of the original non-filtered assembly containing all the genes and isoforms was measured. Therefore, additional filtering steps reduced the presence of putative paralogues and duplicated sequences in the assembly. A transcriptome is considered to be of high quality if it contains over 80% of BUSCO genes [38,45], so our data indicate good coverage of the generated non-redundant transcriptome assembly of *V. speciosa* since there was a high recovery of conserved orthologues from eukaryotes in general and from plants in particular. However, the alignment against Embryophyta core genes was at the lower end of what it is considered a good quality transcriptome. This might be because we analyzed only the sporophyte phase, whereas complete general species transcriptomes should include sequencing from multiple tissues [45,46]. Thus, taken together, BUSCO results and transcripts contiguity measures (Table 2) indicate that we have an acceptable sporophyte transcriptome assembly. In addition, to the best of our knowledge, the *V. speciosa* sporophyte transcriptome is one of the most complete fern transcriptome hitherto published. Transcriptome assemblies from combined fern gametophyte and sporophyte tissues of *Polypodium amorphum* [42] and *Ceratopteris richardii* [44] recovered 71% of complete conserved embryophyta genes, whereas this value was only 53% for *Lygodium japonicum* (Thunb.) Sw. [42,47].

### 3.3. Coding Regions Identification and Functional Annotation

TransDecoder determined that the total number of protein-coding transcripts among the final non-redundant transcripts was 29,220. Approximately 59.8% of the coding sequences were complete, 21.4% were 5′ partial, 10.4% were 3′ partial, and 8.4% were internal.

BLAST analysis performed using BLAST2GO assigned valid hits to 17,706 transcripts, while 18,724 remained without BLAST assignation (Figure S1). The distributions of the BLAST result parameters show a high proportion of low *e*-values (Figure S2) and high similarity values (Figure S3). In addition, the species distribution shows *Arabidopsis thaliana* as the most abundant species with more than 66% of the BLAST results (Figure S4). 

**Figure 2:**
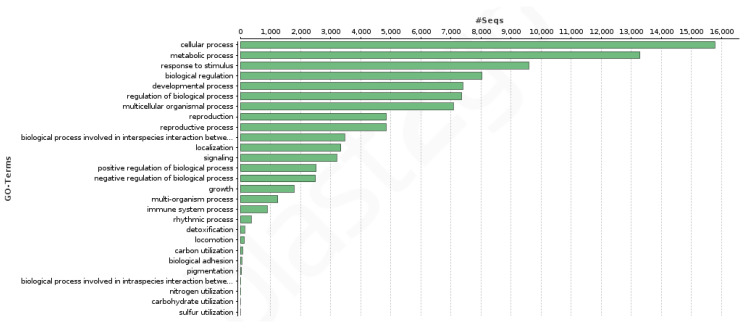
Distribution of the GO terms in the second GO hierarchical level for the biological process category.

**Figure 3:**
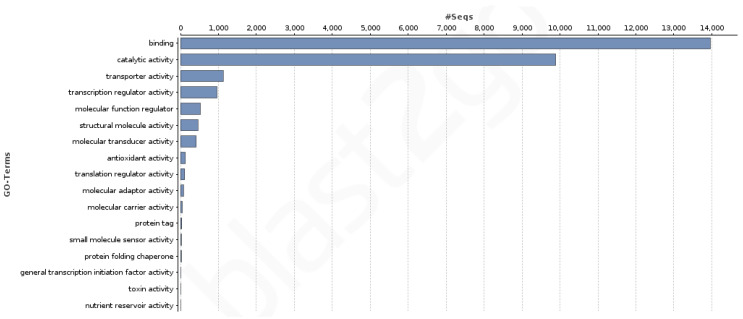
Distribution of the GO terms in the second GO hierarchical level for the molecular function category.

A total of 19,539 transcripts showed at least one of the 7362 GO terms assigned to the transcriptome: 3871 from the biological process category, 2726 from the molecular function category, and 765 from the cellular component category. Regarding the KEGG terms count, 6547 transcripts showed at least one of the 1359 KEGG terms assigned in the transcriptome. Figure 2 and Figure 3 show the GO terms distribution in the second GO hierarchical level for the biological process and the molecular function categories. The developmental, reproduction, reproductive processes, and growth as well as the binding activity (which includes transcription factors) are notorious.

### 3.4. Transcript Abundance Analysis and Study of Expressed Genes

The 17,706 transcripts with a BLAST description were ordered from highest to lowest TPM values. We searched further into the descriptions of the top 1000 transcripts with the highest TPM values (Table S1). The maximum TPM value was 9220.64 and the 1000th transcript had a TPM value of 88.04. Among these transcripts with the highest TPM values, an important percentage represented genes involved in chloroplastidial (37.6%) and mitochondrial (17.2%) functions. The list also includes ribosomal and translational-apparatus genes (10.5%), cytoskeleton structural constituent genes (4.4%) and histone genes (2.5%). Nevertheless, 28.8% of the top 1000 transcripts classified by TPM values represented genes involved in plant growth and development, with 10% of them involved in the regulation of vegetative to reproductive phase transition. Identification of transcripts homologous to genes involved in the vegetative to reproductive transition and in the alternation between the sporophytic and the gametophytic phases is crucial for the study of conservation genetics of ferns in general and specifically for *V. speciosa*. In addition, this fern is an endangered species characterized by the existence of populations where the gametophyte can survive in the absence of the sporophyte by vegetative growth [9]. Thus, Tables S2 and S3 contain those transcripts that are homologous to genes involved in the vegetative to reproductive transition in flowering plants (487 transcripts) and those homologous to genes involved in the genetic control of plant development in flowering plants (5968 transcripts). Additionally, Table S4 lists the transcripts representing transcription factors (1084 transcripts), with special interest in those that regulate plant development such as MADS box or Homeobox genes [48,49].

We prospectively checked the list of the 1000 most expressed transcripts and we found promising clues for future research: (a) genes involved in the vegetative growth in flowering plants were abundant within this list; (b) there is a remarkable presence of transcripts from genes involved in the synthesis of glucomannans, which constitute the type III primary cell wall in vascular plants and that are exclusively reported in some fern species [50,51]; (c) 43.1% of the transcripts were related to stress response (17.3% defense; 9.7% water deprivation conditions; 16.1% abiotic stress, especially salt and oxidative stress, both clearly related to drought and hydric stress). This latter observation is relevant since the sporophyte of this species has little control over water loss [9,10,11] and a requirement for constantly humid and warm winter conditions that restricts the species to extreme low light environments [9,10,11].

In addition to the annotated transcripts, 18,724 transcripts remained anonymous (not annotated using BLASTx). The latter had TPM values higher than 47.73 among the 1000 most expressed transcripts. Furthermore, the first four transcripts showed TPM values above the highest value among annotated transcripts and 449 transcripts had TPM higher than 88.11 (lowest value among the top 1000 blasted). Seventy-nine of these transcripts had GO term annotation. In fact, 2816 of the total 18,724 anonymous transcripts showed an assigned GO term. Therefore, there is a number of potentially important hitherto unknown transcripts that merit future research—especially those with high TPM values.

### 3.5. Homology and Orthology Prediction

A total of 217,519 proteins (82.5%) from eight plant transcriptomes (including *V. speciosa*) were assigned to 26,875 orthogroups (Table S5). Therefore, our results indicate correct species sampling since more than 80% proteins were assigned [35,36]. In addition, the percentages of assigned proteins per species were more than 80% for most of them (Table 4). However, the percentages of proteins that were assigned to orthogroups in the case of the two mosses *Ceratodon purpureus* and *Physcomitrella patens* were 70% and 78%, respectively (Table 4). This is probably due to evolutionary divergence since vascular plants are quite distantly related to these two moss species. In fact, a more restricted analysis comprising only vascular plants or just pteridophytes (lycophytes and monilophytes) resulted in a higher number of transcripts assignation with all species surpassing 80% assignation. Nevertheless, we assumed the loss of mosses information on behalf of a deeper analysis in ferns.

**Figure 4:**
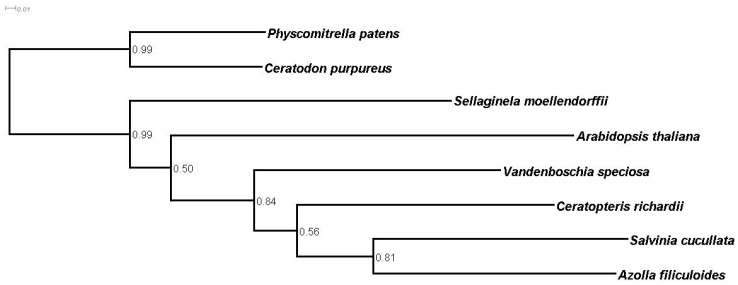
Phylogenetic tree based on single-copy orthologues. The tree was rooted with the non-vascular species (*C. purpureus* and *P. patens*). Numbers are bootstrap values for individual nodes.

A phylogenomic analysis based on the substitution rates of single-copy orthologues resulted in the species tree shown in Figure 4. The tree is congruent with current phylogeny and with those obtained in other fern genomic projects [42,44,52], so that in vascular plants, there is a basal dichotomy, separating lycophytes (*Sellaginela moellendorffii*) from the euphylophytes, which diverge in two major clades—the monilophytes or ferns (represented here by four species) and the spermatophytes or seed plants (represented in this paper by *Arabidopsis thaliana*) [1]. Among the four classes composing Monilophyte, the class Polypodiopsida (leptosporangiate ferns) includes most extant ferns, with more than 80% of about 10,500 fern species. Three leptosporangiate orders, Salviniales (heterosporous ferns, represented in this paper by *Azolla filiculoides* and *Salvinia cucullata*), Cyatheales (tree ferns) and Polypodiales (polypods; represented in this paper by *Ceratopteris richardii*), form the large monophyletic clade of core leptosporangiates [2]. The rest of the leptosporangiate species are included within the orders Osmundales, Hymenophyllales (represented here by *V. speciosa*), Gleicheniales and Schizaeales. The species tree based on single-copy orthologues of Figure 4 agree with this classification and show fern species forming a group in which heterosporous ferns are differentiated from the homosporous ferns, with *V. speciosa* as a lineage that separates early from core leptosporangiate ferns [53,54,55,56,57,58,59]. Homosporous ferns also differ from heterosporous ferns in that the former have extremely large genomes with an average genome size of 12 Gb [60], whereas the latter have smaller genomes secondarily reduced (0.75 Gb in the case of *Azolla filiculoides* and 0.25 Gb in the case of *Salvinia cucullata*, for example) [52]. Polyploidization has been suggested as a major factor contributing to high chromosome numbers and large genomes in ferns [61,62]. In this context, *V. speciosa* is an allotetraploid species with 2*n* = 144 chromosomes, which in part would explain its large genome (1C = 10.52 Gb) [4,5,6,7,8].

Monilophytes represent a phylogenetically important branch within the land plant lineage. However, there are few fern genomic studies, probably due to the huge sizes of fern genomes, especially in the case of leptosporangiate ferns (most ferns). In fact, there are only two complete fern genome assemblies, both belonging to two heterosporous ferns which have much smaller genomes [52] and some partial genome assemblies from homosporous ferns [61,63]. On the other hand, there is an increasing number of studies that are based on transcriptome assemblies, most of them obtained only from the vegetative tissue and a few from combined gametophyte and sporophyte tissues [42,44,60,64]. Nevertheless, fern genomes and transcriptomes have been insufficiently explored and the data are still scarce and fractionated. Therefore, enlarging ferns genomic resources is an essential task [65,66], supported in this paper by the addition of the *V. speciosa* transcriptome, a species that has biological and ecological relevance. The progressive accumulation of more information about the genomes of ferns and organisms from other basal branches of land plants will favor more accurate phylogenetic analyses [52] as well as insights on the evolution of crucial regulators in the control of plant growth and developmental processes [42,44,52,67,68]. In this sense, Geng et al. [44] analyzed the evolutionary history of the GRAS domain proteins, which form an important superfamily of regulatory proteins in shoot and root development, stem cell homeostasis, light and hormone signaling, responses to biotic and abiotic stresses, and symbiosis with microorganisms [69], and from which we found 22 transcripts within the *V. speciosa* sporophyte transcriptome. Similarly, Sigel et al. [42] also contributed to the study of several interesting gene families (phototropins, terpene synthases, and type II MADS-box genes) and our work provides more data for similarly promising comparative studies that would include data from a basal leptosporangiate fern.

A total of 5102 of the orthogroups (19%) were shared among the eight species. Of these, 101 were single-copy orthogroups, i.e., orthogroups with exactly one protein from each species (Table S5). A total of 47,903 proteins (18%) were potentially species-specific, grouped in 12,163 species-specific orthogroups (Table S5). This high number of potentially species-specific proteins is congruent with the diversity of the compared species.

A total of 23,964 out of the 29,220 identified coding sequences of *V. speciosa* (82%) were assigned to one of the 11,195 orthogroups (Table 4). Thus, the remaining 5,256 sequences might be single-copy species-specific coding sequences. In addition, 12.65% (3,696) of *V. speciosa* coding sequences represented species-specific ones grouped in 1,384 orthogroups (Table 4). This is interesting as it may reflect an important percentage of lineage-specific genes as it was found in other ferns [42,44,52] However, as pointed out by Sigel et al. [42] all these studies also broadly support the notion that gene family identity across all land plants is substantially conserved [67,68,70,71]. Table 5 shows shared orthogroups between *V. speciosa* and the rest of species.

The orthology relationships (one-to-one; one-to-many, etc.) between *V. speciosa* and the rest of species were also analyzed (Table S6). The results shown in Table 5 and Table S6 are congruent with phylogenetic distances. However, the quantitative comparisons between *V. speciosa* and the rest of species are taken cautiously here because the protein number in *V. speciosa* is underestimated since we have only considered the genes that are expressed during the sporophytic phase of this species. Notwithstanding, different studies have revealed significant overlapping patterns of gene expression between gametophyte and sporophyte phases, as in mosses [72], but contrary to what occurs in seed plants [73]. For example, 68.2% of the genes are expressed in both phases in *Ceratopteris richardii* [44], 85% in *Lygodium japonicum* [47] and 97.7% in the case of *Polypodium amorphum* [42]. Additionally, it is interesting to highlight the disparity in ploidy levels and polyploidization ages between the different lineages compared here.

We therefore focused our analysis specifically in those orthogroups for which *V. speciosa* had an enriched number of transcripts. Among these orthogroups, transcripts representing genes encoding pentatricopeptide repeat-containing proteins were especially over-represented (1787), although they probably belong to a lower number of genes. Notwithstanding, Li et al. [52] found that the pentatricopeptide repeat (PPR) family is the largest gene family found in the *Azolla* (over 2000 PPR proteins) and *Salvinia* (over 1700 PPR proteins) genomes. They found that the large repertoire of PPRs correlates well with the extensive RNA editing observed in the organellar genomes of Salviniales [52], a phenomenon also observed in *V. speciosa* plastome [74]. The list with an enriched number of genes in *V. speciosa* includes some other genes with interesting products, such as proteins involved in disease resistance, chromatin organization, several classes of transcription factors, different proteins controlling cell division, cell differentiation, cell wall formation, plant growth and plant development, proteins regulating dormancy or response to water deprivation, spliceosomal complex components or enzymes involved in lignin and mannan biosynthesis.

### 3.6. Blastx Comparison between V. speciosa and A. thaliana Transcriptomes

tBlastx of the *A. thaliana* transcriptome (current version, v11) against the *V. speciosa* transcriptome gave 456,516 significant hits at a 10^−5^ evalue cutoff (Table S7). When considering only the top blast result for each *V. speciosa* transcript, these corresponded to 10,289 transcripts out of the 48,293 that form the *A. thaliana* transcriptome (21.31%) and 10,294 *V. speciosa* transcripts out of the 17,706 annotated transcripts of that species transcriptome (58.14%)—28.26%% if we consider all the 36,430 transcripts that form the whole *V. speciosa* transcriptome that we assembled as reference (annotated and with no known annotation). Such a large number of shared transcripts reflect the completeness of our transcriptome, whereas the transcripts of the *A. thaliana* transcriptome that do not appear in the *V. speciosa* transcriptome are very likely to be transcripts usually expressed in specific *A. thaliana* tissues, or developmental/physiological states not covered in our material. On the other hand, the transcripts present in our fern transcriptome but not in *A. thaliana*’s are probably fern specific genes. To that, we have to include the transcripts of proteins that have diverged too much as to be picked by our blast search. In fact, the mean percentage identity between the blast high-scoring parts of the sequences of both species is 47.68%, which reflects the evolutionary distance between both species.

## 4. Conclusions

High-throughput RNA sequencing has emerged as a powerful tool for gene identification and gene expression analyses. In this work, we characterized and analyzed the transcriptome of the sporophyte (one of the two phases) of an endangered fern species, *V. speciosa*. This is the first step towards understanding gene expression in a species that presents extreme ecological restrictions and biological particularities. Our study has unveiled outstanding clues about the transcriptomics of the adaptative response to hydric stress, the genetic control of sporophyte growth and development and the alternation between the vegetative and reproductive phases of this species. These results provide an important resource for further RNA sequencing studies of other fern transcriptomes, including that of the gametophyte of this species, to better understand the conservation genetics of ferns in general and, specifically, of *V. speciosa*.

## Figures and Tables

**Table 1:** Sequencing statistics.

	Raw Data	after Quality Trimming
Number of paired-end reads	66.3 million	65.2 million
Number of bases	6700 million	6590 million

**Table 2:** Assembly statistics.

	Before Filtering	After Filtering
Total transcripts	84,759	36,430
Percent GC	45.18	45.18
Contig N50 (bp)	1955	2085
Contig N70 (bp)	1332	1511
Contig N90 (bp)	479	729
Ex90N50 (bp)	2039	2299
Number transcripts corresponding to the Ex90 peak	14,645	21,543
Size of the smallest contig (bp)	201	201
Size of the largest contig (bp)	13,225	13,224
Number of contigs greater than 1 Kb long	35,801	20,532
Number of contigs greater than 10 Kb long	18	12
Median contig length (bp)	722	1197
Average contig (bp)	1,144.86	1,437.37
Total number of assembled bases	97,037,551	52,363,571

**Table 3:** The table lists the number of proteins from the Swiss Prot database on which the *V. speciosa* transcripts align along a percentage of their length.

	Before Filtering		After Filtering	
Percentage Intervals	Number of Proteins *	Accumulated Number of Proteins **	Number of Proteins *	Accumulated Number of Proteins **
91–100	3301	3301 (>90%)	3196	3196 (>90%)
81–90	1330	4631 (>80%)	1352	4548 (>80%)
71–80	916	5547 (>70%)	878	5426 (>70%)
61–70	646	6193 (>60%)	569	5995 (>60%)
51–60	551	6744 (>50%)	449	6444 (>50%)
41–50	586	7330 (>40%)	448	6892 (>40%)
31–40	538	7868 (>30%)	381	7273 (>30%)
21–30	506	8374 (>20%)	302	7575 (>20%)
11–20	411	8785 (>10%)	225	7800 (>10%)
1–10	90	8875 (>1%)	51	7851 (>1%)
TOTAL	8875	8875	7851	7851

* Number of proteins that each match a *V. speciosa* transcript in a percentage of their lengths comprised in the indicated interval; ** Number of proteins that each match a *V. speciosa* transcript in a percentage of their lengths above the percentage indicated in brackets.

**Table 4:** Per species statistics of the Orthofinder analysis.

	Mosses		Lycophyte	Seed Plant	Leptosporangiate Ferns			
Statistics	*Cp*	*Pp*	*Sm*	*At*	*Vs*	*Cr*	*Af*	*Sc*
Number of proteins	40,806	38,354	22,285	48,359	29,220	44,668	20,203	19,779
Number of proteins in orthogroups	31,747	26,769	20,136	44,174	23,964	35,963	17,948	16,818
Number of unassigned proteins	9059	11,585	2149	4185	5256	8705	2255	2961
Percentage of proteins in orthogroups	77.8	69.8	90.4	91.3	82.0	80.5	88.8	85.0
Percentage of unassigned proteins	22.2	30.2	9.6	8.7	18.0	19.5	11.2	15.0
Number of orthogroups containing species	12,351	11,499	9586	12,003	11,195	12,715	9915	9882
Percentage of orthogroups containing species	46.0	42.8	35.7	44.7	41.7	47.3	36.9	36.8
Number of species-specific orthogroups	1654	813	1461	4018	1384	2207	344	282
Nº of proteins in species-specific orthogroups	6849	2540	6545	18,005	3696	8205	1142	921
% of proteins in species-specific orthogroups	16.8	6.6	29.4	37.2	12.6	18.4	5.7	4.7

Cp: *C. purpureus*; Pp: *P. patens*; Sm: *S. moellendorffii*; At: *A. thaliana*; Vs: *V. speciosa*; Cr: *C. richardii*; Af: *A. filiculoides*; Sc: *S. cucullata*.

**Table 5:** Number of shared orthogroups between *V. speciosa* and the rest of species.

	*A. filiculoides*	*C. purpureus*	*C. richardii*	*P. patens*	*S. cucullata*	*S. moellendorffii*	*V. speciosa*
*A. thaliana*	6905	6921	7275	6954	6882	6753	7243
*A. filiculoides*		7169	8675	7188	8448	6901	8028
*C. purpureus*			7576	10339	7145	7072	7433
*C. richardii*				7576	8665	7309	8916
*P. patens*					7167	7070	7443
*S. cucullata*						6901	8052
*S. moellendorffii*							7198

## Supplementary Materials

The following are available online at https://www.mdpi.com/article/10.3390/genes12071017/s1, Figure S1: Tag distribution; Figure S2: E-value distribution; Figure S3: Sequence similarity distribution; Figure S4: Species distribution. Table S1: Top 1000 transcripts with highest TPM values. Table S2: Transcripts that are homologous to genes involved in the vegetative to reproductive transition in flowering plants. Table S3: Transcripts that are homologous to genes involved in the genetic control of plant development in flowering plants. Table S4: Transcripts representing transcription factors. Table S5: Overall statistics of the Orthofinder analysis for eight land plant species. Table S6: Orthology relationships between *V. speciosa* and seven land plant species. Table S7: tBlastx results of the *A. thaliana* transcriptome against the *V. speciosa* transcriptome.
