# Supplementary material for: De Novo Sporophyte Transcriptome Assembly and Functional Annotation in the Endangered Fern Species Vandenboschia speciosa (Willd.) G. Kunkel
Source: Genes (Basel). 2021 Jun 30;12(7):1017. doi: 10.3390/genes12071017 (PMC8304985; doi:10.3390/genes12071017)
Supplement: Supplementary file 1 [file genes-12-01017-s001.zip › genes-1262071-supplementary/Supplementary files/Table S5.pdf]

Table S5. Overall statistics of the Orthofinder analysis for eight land plant species

Statistic	Value
Number of species	8
Number of proteins	263674
Number of proteins in orthogroups	217519
Number of unassigned proteins	46155
Percentage of proteins in orthogroups	82.5
Percentage of unassigned proteins	17.5
Number of orthogroups	26875
Number of species-specific orthogroups	12163
Number of proteins in species-specific orthogroups	47903
Percentage of proteins in species-specific orthogroups	18.2
Mean orthogroup size	8.1
Median orthogroup size	4.0
Number of orthogroups with all species present	5102
Number of single-copy orthogroups	101
