# Supplementary material for: De Novo Sporophyte Transcriptome Assembly and Functional Annotation in the Endangered Fern Species Vandenboschia speciosa (Willd.) G. Kunkel
Source: Genes (Basel). 2021 Jun 30;12(7):1017. doi: 10.3390/genes12071017 (PMC8304985; doi:10.3390/genes12071017)
Supplement: Supplementary file 1 [file genes-12-01017-s001.zip › genes-1262071-supplementary/Supplementary files/Table S6.pdf]

Table S6. Upper part. Number of genes of *V. speciosa* that have homologous genes in each of the other seven species analyzed. **Lower part.** Number of genes of each species having homologous genes in *V. speciosa*.

Species	One-to-one*	One-to-many*	Many-to-one*	Many-to-many*	**Total**
Arabidopsis thaliana	1539	2568	3009	6716	**13832**
Azolla filiculoides	4139	1387	6536	3046	**15108**
Physcomitrella patens	1886	2152	3842	7070	**14950**
Salvinia cucullata	4370	1140	7008	2669	**15187**
Sellaginela moellendorffii	3254	865	7244	2926	**14289**
Ceratodon purpureus	2184	1812	5101	5767	**14864**
Ceratopteris richardii	2890	3518	4467	6259	**17134**

	A. thaliana	*A. filiculoides*	*P. patens*	*S. cucullata*	*S. moellendorffii*	*C. purpureus*	*C. richardii*
One-to-one*	1539	4139	1886	4370	3254	2184	2890
One-to-many*	1078	2549	1384	2711	2594	1734	1732
Many-to-one*	9714	3500	6490	2780	2420	6258	9778
Many-to-many*	9738	2786	7641	2343	2545	6803	6737
**Total**	**22069**	**12974**	**17401**	**12204**	**10813**	**16979**	**21137**

*One-to-one: One gene in species A is homologous to other gene in species B
One-to-many: One gene in species A is homologous to several paralogous genes in species B
Many-to-one: Several paralogous genes in species A are homologous to just one gene in species B
Many-to-many: Several paralogous genes in species A are homologous to several paralogous genes in species B
