# Supplementary figures and images for: De Novo Sporophyte Transcriptome Assembly and Functional Annotation in the Endangered Fern Species Vandenboschia speciosa (Willd.) G. Kunkel
Source: Genes (Basel). 2021 Jun 30;12(7):1017. doi: 10.3390/genes12071017 (PMC8304985; doi:10.3390/genes12071017)

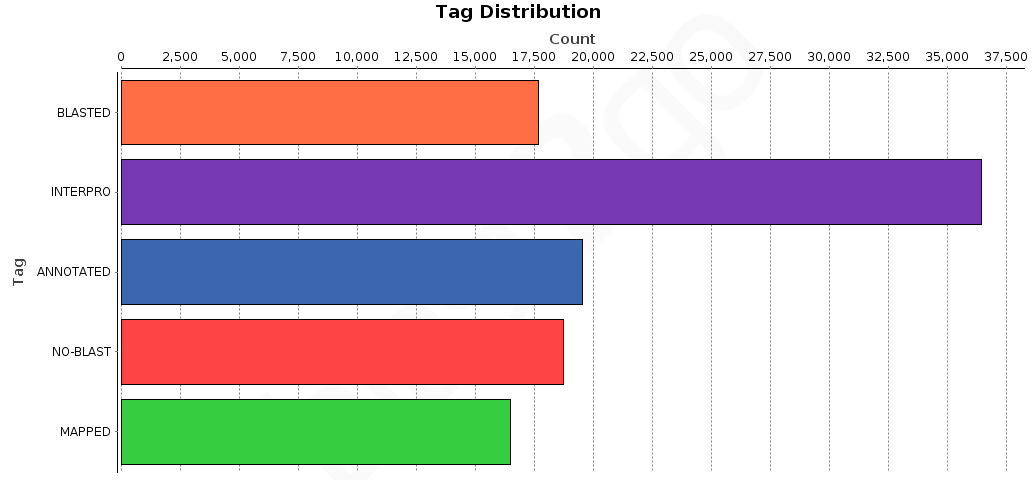

Supplement: Supplementary file 1 [file genes-12-01017-s001.zip › genes-1262071-supplementary/Supplementary files/Figure S1.jpg]

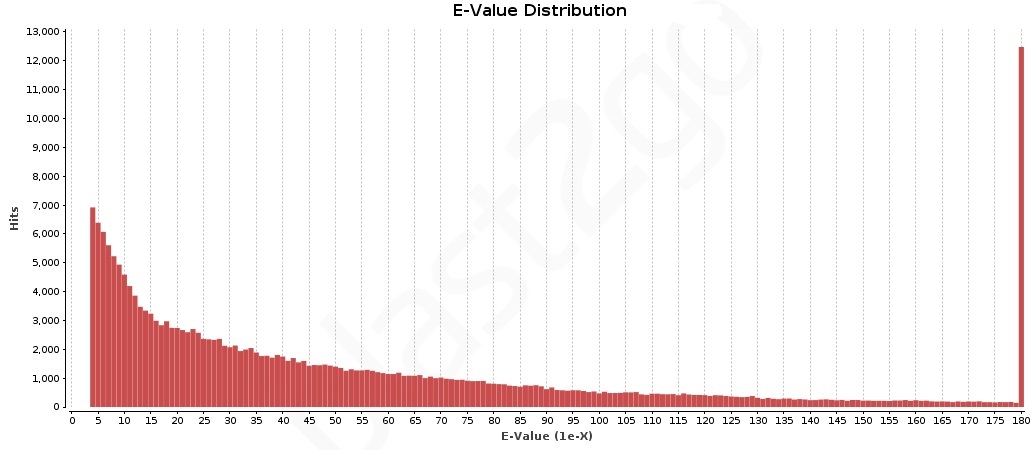

Supplement: Supplementary file 1 [file genes-12-01017-s001.zip › genes-1262071-supplementary/Supplementary files/Figure S2.jpg]

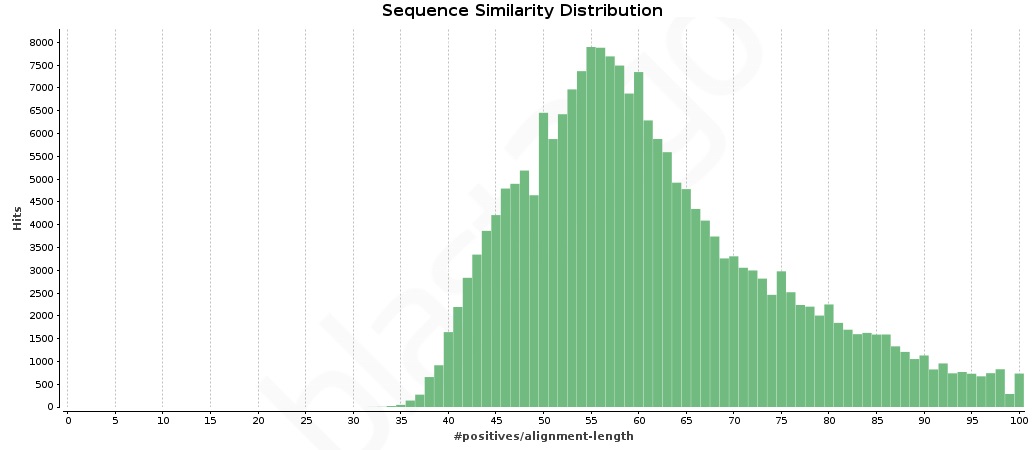

Supplement: Supplementary file 1 [file genes-12-01017-s001.zip › genes-1262071-supplementary/Supplementary files/Figure S3.jpg]

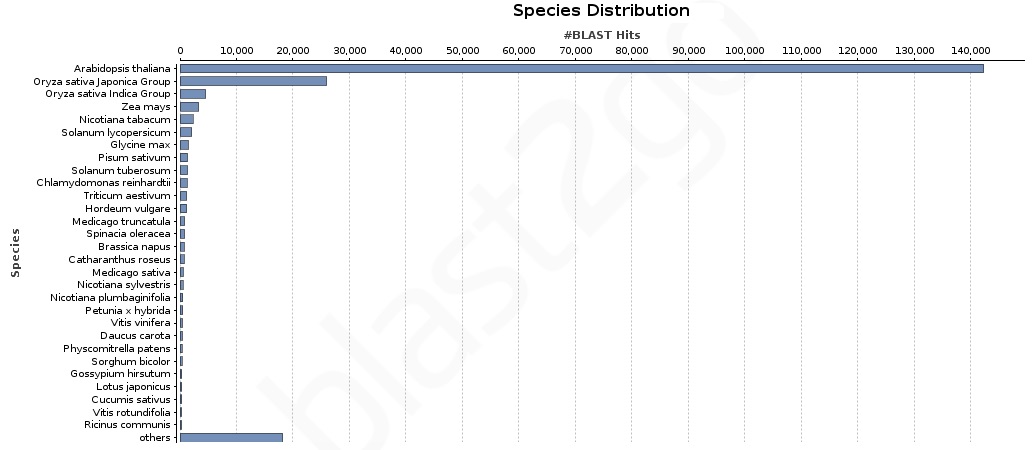

Supplement: Supplementary file 1 [file genes-12-01017-s001.zip › genes-1262071-supplementary/Supplementary files/Figure S4.jpg]
